# Predictors of lateral lymph node metastasis and skip metastasis in patients with papillary thyroid microcarcinoma

**DOI:** 10.3389/fendo.2024.1392247

**Published:** 2024-07-02

**Authors:** Jee Hee Yoon, Ji Yong Park, A Ram Hong, Hee Kyung Kim, Ho-Cheol Kang

**Affiliations:** ^1^ Department of Internal Medicine, Chonnam University Medical School, Gwangju, Republic of Korea; ^2^ Division of Endocrinology and Metabolism, Department of Internal Medicine, Chonnam University Hwasun Hospital, Gwangju, Republic of Korea

**Keywords:** papillary thyroid microcarcinoma (PTMC), lymph node (LN), extra-thyroidal extension (ETE), upper lobe, non-parallel shape, multifocality

## Abstract

**Background:**

Papillary thyroid microcarcinoma (PTMC) is characterized by its favorable prognosis and potential for active surveillance (AS) as a management option. However, the presence of cervical lymph node (LN) metastasis, especially lateral LN metastasis, significantly impacts management and prognosis. Previous studies have focused on post-surgery risk factors for cervical LN metastasis. This study aims to identify predictors of lateral LN metastasis by analyzing pre-operative ultrasonographic findings alongside clinicopathological factors.

**Methods:**

A retrospective review of medical records was conducted for patients with PTMC who underwent surgery at Chonnam National University Hwasun Hospital between 2004 and 2013. This is a case–control study that compares patients with lateral LN metastasis (N1b) to age- and sex-matched patients without LN metastasis (N0). Subgroup analysis was performed to evaluate risk factors of skip metastasis.

**Results:**

The study included 90 patients with PTMC with lateral LN metastasis (N1b) and 268 age- and sex-matched patients without LN metastasis (N0). The mean age was 49.3 years, and female patients were dominant in both groups. Structural recurrences of 4.4% (4/90) were observed only in the N1b group. The N1b group exhibited a higher frequency of upper lobe tumor location compared to the N0 group (38.9% vs. 16.0%, *p* < 0.001). There was no significant difference in the locations with the presence of invasion to adjacent organs. A higher proportion of non-parallel shape was observed in the N1b group than the N0 group (80.0% vs. 66.0%, *p* = 0.013). There were no differences in echogenicity, sonographic feature, margin, and AP diameter of the thyroid gland between the two groups. In multivariate analysis, independent risk factors for lateral LN metastasis included extrathyroidal extension, multiplicity, upper lobe tumor location, and non-parallel shape. Skip metastasis in patients with PTMC was associated with upper lobe tumor location.

**Conclusion:**

Detailed ultrasound examinations, evaluating tumor location, number, orientation, and the presence of ETE, are crucial in accurately predicting lateral LN metastasis especially when primary tumor was in the upper lobe to avoid missing skip metastasis. These evaluations can help guide the decision between AS and immediate surgery in patients with PTMC.

## 1 Introduction

The indolent nature and excellent prognosis of papillary thyroid microcarcinoma (PTMC) have contributed to the emergence of active surveillance (AS) as a viable treatment approach for low-risk thyroid cancer (1). AS for low-risk PTMC was introduced in Japan, and the American Thyroid Association guideline also recommended AS as an applicable management strategy in selected cases of low-risk thyroid cancer (2). A Korean study revealed a good prognosis, including 1.5% recurrence rate during a median follow-up of 7.7 years and 0.1% distant metastasis rate among 8,808 patients with PTMC (3). However, physician- and patient-related barriers to accepting AS in patients with PTMC still exist, and one of the main barriers is the fear of cancer progression, even though the risk of disease progression is low. Therefore, appropriate candidate identification for AS, especially the exclusion of cases with high risks of disease progression, is important.

Many studies have evaluated the prognostic factors of PTMC, including old age, male gender, larger tumor size, extrathyroidal extension (ETE), lymph node (LN) metastasis, multifocality, BRAF mutation, and coexistence of chronic thyroiditis, with varying or inconclusive results (4–8). The majority of prognostic factors can be ascertained through postoperative pathological results, which makes their utilization challenging for patients under consideration for AS. However, all patients diagnosed with PTMC undergo ultrasonography (US) before the AS decision to identify high-risk features, such as invasion and LN metastasis. Distant metastasis in patients with PTMC was rarely observed, but it could be fatal and all patients with PTMC with distant metastasis had synchronous cervical LN metastasis at the time of the first diagnosis (3). In particular, lateral LN metastasis is a prognostic factor of recurrence in patients with PTMC (9). Most of the lateral LN metastases in patients with PTMC progress sequentially from the initial central LN metastasis to the ipsilateral lateral LN metastasis (10). However, skip metastasis with a discontinuous lymphatic spread pattern has been reported in up to 21.8% of cases and PTMC (less than 1 cm) is a risk factor of skip metastasis among patients with papillary thyroid carcinoma (PTC) (11, 12).

Hence, the primary objective of this study is to assess potential risk factors associated with the development of lateral LN metastasis in patients with PTMC. This will be achieved by analyzing US findings in conjunction with clinicopathological factors among patients with lateral LN metastasis (N1b) and comparing them to age- and sex-matched patients without LN metastasis (N0). Additionally, we analyzed risk factors for skip metastasis in patients with PTMC with lateral LN metastasis.

## 2 Materials and methods

### 2.1 Patient population

**Figure 1:**
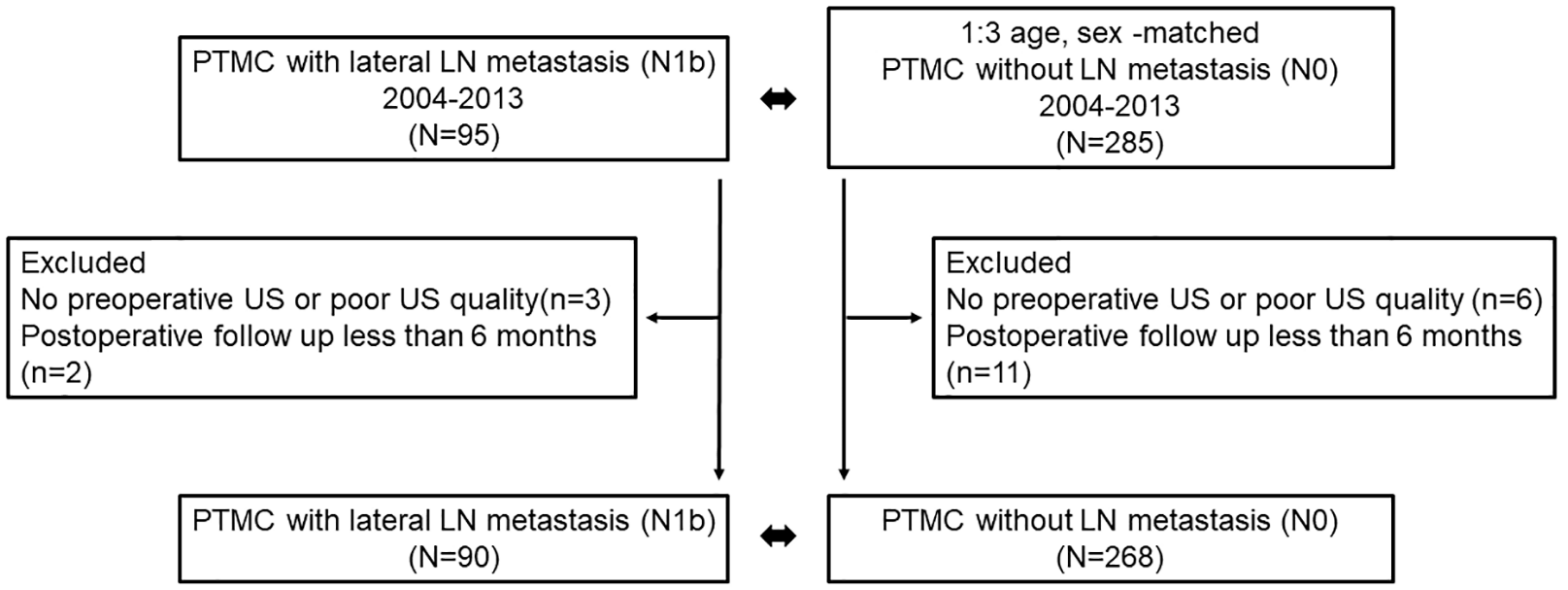
Flow chart of study population.

This study is a retrospective, age- and sex-matched case–control cohort study where we reviewed medical records of patients diagnosed with PTMC who underwent surgery at Chonnam National University Hwasun Hospital between 2004 and 2013. The eligibility criteria included patients with PTMC with lateral LN metastasis (N1b) at the first surgery, resulting in the identification of 95 patients. Propensity score matching (PSM) analysis was used to match patients with PTMC with lateral LN metastasis (N1b) and those without LN metastasis (N0) based on age and sex as a confounding factor, with a matching ratio of 1:3. We utilized the “Matchit” package in R software (version 3.3.3) for PSM analysis, and 285 patients were enrolled as controls. Among the 95 patients with lateral LN metastasis, we excluded three patients who had no preoperative US and poor US quality, as well as two patients with insufficient follow-up periods of less than 6 months. In the control group without LN metastasis, we excluded 6 patients with no preoperative US and poor US quality, and 11 patients with insufficient follow-up periods of less than 6 months. Finally, we included and analyzed 90 patients with lateral LN metastasis (N1b) and 268 patients without LN metastasis (N0) to identify risk factors for lateral LN metastasis in patients with PTMC (**Figure 1**).

Informed consent was waived due to the retrospective nature of the study, and the Institutional Review Board of Chonnam National University Hwasun Hospital (No. CNUHH-2020–271) approved this study.

### 2.2 Evaluation of clinical and histopathological factors

To assess risk factors for lateral LN metastasis, we collected and analyzed baseline clinical characteristics and pathologic results. ETE included both minimal and gross invasion in pathologic results. Skip metastasis and stepwise metastasis were defined as lateral LN metastasis without central LN metastasis and lateral LN metastasis with central LN metastasis, respectively. Thyroid-stimulating hormone (TSH) levels were measured using Elecsys and Cobase analyzer kits (Roche Diagnostics, GmbH, Mannheim, Germany), with laboratory reference ranges of 0.4–4.48 mIU/L. At the last follow-up point, structural recurrence was defined as evidence of structural or functional disease, regardless of serum thyroglobulin (Tg) level or anti-Tg antibody (2).

### 2.3 Evaluation of preoperative thyroid image findings

Preoperatively, thyroid US was performed to assess the thyroid gland and neck. Gray-scale static US images of the thyroid and nodules were acquired using either the Logiq9 system (GE Medical System, Milwaukee, WI, USA) or ACUSON Antares system (Siemens Medical Solutions, Malvern, PA, USA) with a linear high-frequency probe (5–13 MHz). Two expert endocrinologists (K.C.H. and K.H.K.) conducted the US imaging. Following a training session with randomly selected US images, consensus criteria for the US images were defined, and one endocrinologist (P.J.Y.) reviewed the images. In cases where multiple suspicious nodules were present, the dominant nodule was determined, based on its aggressive nature as determined by the pathologic result, or if aggressiveness was not discernible, the largest nodule was selected as the dominant nodule.

### 2.4 US for thyroid nodule (main thyroid tumor)

Each nodule was evaluated using at least two US images, including the transverse and longitudinal planes. Tumor location was classified into upper, mid, and lower categories (13). For the assessment of invasion, tumor location 2 was categorized into various groups, including intra-thyroidal, adjacent to the anterolateral capsule, adjacent to the post-capsule, adjacent to the trachea, gross ETE to the strap muscle, gross ETE to the recurrent laryngeal nerve (RLN), or gross ETE to the trachea (14). The trachea invasion risk stratification (tumor location 3) was divided into three categories based on the angles between the tumor and the trachea: acute angle, right angle, or obtuse angle (15). Nodule composition was categorized as solid, partially cystic, partially solid, or cystic based on the ACR TI-RADS (American College of Radiology Thyroid Imaging, Reporting, and Data System) (16, 17). Nodular echogenicity was classified as marked hypoechoic, mild hypoechoic, isoechoic, or hyperechoic compared to the echogenicity of anterior neck muscles and normal thyroid parenchyma as reference (18). Nodular margin was divided into three categories: smooth, irregular, or ill-defined (16). Nodular orientation was categorized as either parallel or non-parallel (taller than wide shape). Finally, calcifications were divided into four categories: no calcification, punctate echogenic foci, macrocalcification, or rim (peripheral) calcification (16).

### 2.5 US for the thyroid gland (diffuse thyroid disease)

To assess the presence of diffuse thyroid disease (DTD), US findings of the thyroid were evaluated, including echogenicity (normal versus decreased), echotexture (fine or coarse/micronodular), margin (smooth, microlobulated, or macrolobulated), and the anteroposterior (AP) diameter of the thyroid gland (considering 1–2 cm as the normal reference, decreased, or increased) (19, 20). The vascularity of the thyroid was not analyzed in this study due to the lack of available data.

### 2.6 US for lymph nodes

Among patients with lateral LN metastasis, preoperative US findings of suspicious LNs were collected. The LNs were evaluated based on size, cystic change, calcifications, and echogenicity (particularly abnormal hyperechogenicity) (21).

### 2.7 Statistical analysis

The data are presented as mean ± standard deviation or as *n* (%). Continuous variables were analyzed using Student’s *t*-test, while categorical variables were analyzed using the Chi-square test and Mann–Whitney test. Kaplan-Meier analysis with the log-rank test was used to compare recurrence-free survival between the lateral LN metastasis group and the no-LN metastasis group. Clinical response data were dichotomized based on the presence of structural recurrence. Binary logistic regression models were employed to evaluate predictive risk factors for lateral LN metastasis in patients with PTMC. All statistical analyses were conducted using SPSS Statistics, version 28 (IBM, Armonk, NY), and a *p*-value <0.05 was considered statistically significant.

## 3 Results

### 3.1 Clinical and histopathological characteristics between lateral LN metastasis and no-LN metastasis

The study included a total of 90 patients with PTMC with lateral LN metastasis (N1b) and 268 patients without LN metastasis (N0) (**Table 1**). The mean age in both groups was 49.3 years (48.8 years in N1b group and 49.4 years in N0 group, respectively). All patients in the N1b group underwent total thyroidectomy with central neck dissection and therapeutic lateral cervical neck dissection. Sixty-one patients (67.8%) in the N1b group were underwent therapeutic central neck dissection due to central LN metastasis (N1a).

**Table 1:** Comparison of clinicopathological findings.

	PTMC with lateral LN metastasis [N1b] (*n* = 90)	PTMC without lateral LN metastasis [N0] (*n* = 268)	*p*-value
Age (years)	48.8 ± 11.8	49.4 ± 10.6	0.226
Sex (female, %)	67 (74.4)	200 (74.6)	0.973
Preoperative TSH level (mIU/L)	1.87 ± 1.23	1.99 ± 1.46	0.101
Surgical extent (%)			<0.001
Total thyroidectomy	90 (100.0)	158 (59.0)	
Lobectomy	0 (0.0)	110 (41.0)	
RAI (%)	89^a^ (98.9)	21 (23.3)	**<0.001**
Mean dose of RAI (mCi)	173.2 ± 75.9	56.2 ± 55.1	**<0.001**
Pathologic results			
Cancer type			0.654
Classic	88 (97.8)	262 (97.8)	
Follicular variant	2 (2.2)	4 (1.5)	
Tall-cell variant	0 (0.0)	0 (0.0)	
Cribriform morular variant	0 (0.0)	1 (0.4)	
Oncocytic variant	0 (0.0)	1 (0.4)	
Tumor size (cm)	0.7 ± 0.4	0.6 ± 0.3	**0.009**
Bilaterality (%)	24 (26.7)	23 (8.6)	**<0.001**
Multifocality (%)	40 (44.4)	38 (14.2)	**<0.001**
Extrathyroidal extension (%)	23 (25.6)	12 (4.5)	**<0.001**
Lymphovascular invasion (%)	1 (1.1)	0 (0.0)	0.084
Concurrent chronic thyroiditis (%)	30 (33.3)	97 (36.2)	0.624
Distant metastasis	1 (1.1)	0 (0.0)	0.084
Follow-up months	112.4 ± 41.5	94.6 ± 48.5	**<0.001**

PTMC, papillary thyroid microcarcinoma; LN, lymph node; TSH, thyroid-stimulating hormone; RAI, radioactive iodine.

Data are expressed as mean ± standard deviation (SD) or n (%).

a One patient did not receive RAI therapy.

Compared with PTMC without lateral LN metastasis (N0), P<0.05.

Except for one patient who opted not to receive RAI therapy due to personal preference, RAI therapy was performed in the N1b group. In the N0 group, 59.0% underwent total thyroidectomy and 23.3% received RAI therapy. Classic PTC was the most common subtype in both groups, and there was no significant difference in PTC subtypes between the two groups.

The N1b group showed a larger tumor size (0.7 ± 0.4 cm vs. 0.6 ± 0.3 cm, *p* = 0.009) and more aggressive pathological features, including bilaterality (26.7% vs. 8.6%, *p* < 0.001), multiplicity (44.4% vs. 14.2%, *p* < 0.001), and ETE (25.6% vs. 4.5%, *p* < 0.001) compared to the N0 group. There were no significant differences in capsular invasion, lymphovascular invasion, strap muscle invasion, or concurrent chronic thyroiditis [Hashimoto’s thyroiditis (HT)] between the two groups. The N1b group had ipsilateral LN metastasis, except for two cases with contralateral LN metastasis originating from the primary tumor.

**Figure 2:**
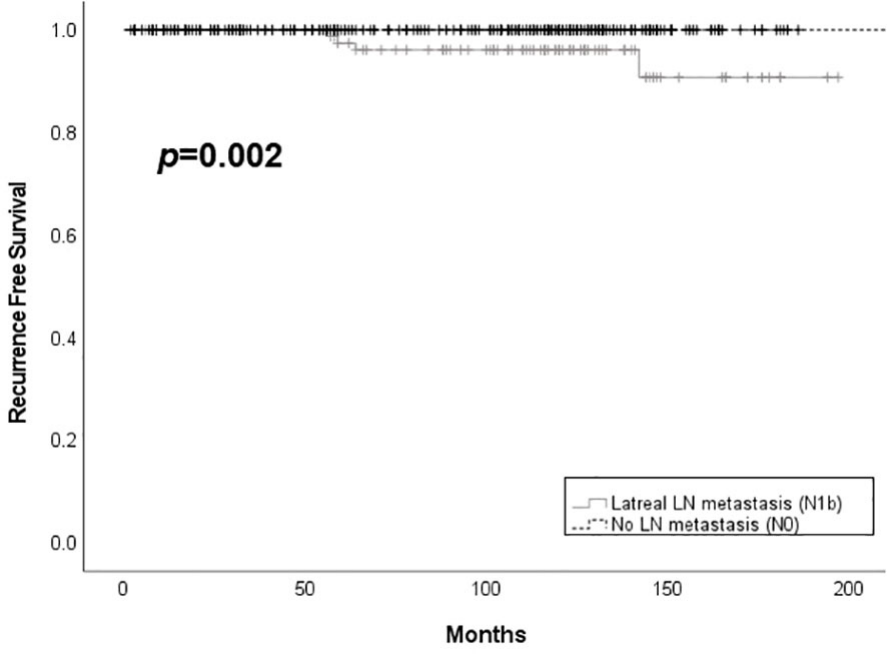
Structural recurrence free survival according to the presence of lateral lymph node metastasis among patients with PTMC. LN, lymph node.

Only one case of metachronous distant metastasis at 82 months after the first surgery was observed in the N1b group. Patients in the N0 group had better structural recurrence-free survival compared to those in the N1b group (*p* = 0.002) (**Figure 2**). Structural recurrence was observed in 4.4% (4/90) of the N1b group. The N1b group had a longer follow-up duration than the N0 group (112.4 ± 41.5 months vs. 94.6 ± 48.5 months, *p* < 0.001).

### 3.2 Ultrasonographic findings according to the presence of lateral LN metastasis

#### 3.2.1 US for dominant thyroid tumor

The tumor locations addressed by US are summarized in **Table 2**. The N1b group showed more frequently upper lobe tumor location compared to the N0 group (38.9% vs. 16.0%, *p* < 0.001). There was no significant difference of the locations with the presence of invasion to adjacent organs including capsule, muscle, trachea, and RLN between N1b and N0 groups. US features of the main thyroid tumor are summarized in **Table 3**. Echogenicity, composition, and margin showed no differences between N1b and N0 groups. Macrocalcification was more frequently observed in the N1b group compared to the N0 group, but the difference was not significant (*p* = 0.053). The N1b group had a higher percentage of non-parallel shape compared to the N0 group (80.0% vs. 66.0%, *p* = 0.013).

**Table 2:** Comparison of tumor locations on ultrasonographic images.

	PTMC with lateral LN metastasis [N1b] (*n* = 90)	PTMC without lateral LN metastasis [N0] (*n* = 268)	*p*-value
Tumor location 1			<0.001
Upper	35 (38.9)	43 (16.0)	
Mid	46 (51.1)	177 (66.0)	
Lower	9 (10.0)	48 (17.9)	
Tumor location 2			0.944
Intra-thyroidal	38 (42.2)	103 (38.4)	
Adjacent to anterolateral capsule	23 (25.6)	67 (25.0)	
Adjacent to post capsule	17 (18.9)	60 (22.4)	
Adjacent to trachea (medial capsule)	9 (10.0)	29 (10.8)	
Gross ETE to strap muscle	2 (2.2)	7 (2.6)	
Gross ETE to RLN	1 (1.1)	1 (0.4)	
Gross ETE to trachea	0 (0.0)	1 (0.4)	
Tumor location 3 (trachea)			0.929
Not adjacent to trachea	81 (90.0)	237 (88.4)	
Acute angle	8 (8.9)	27 (10.1)	
Right angle	0 (0.0)	1 (0.4)	
Obtuse angle	1 (1.1)	3 (1.1)	

PTMC, papillary thyroid microcarcinoma; LN, lymph node; ETE, extra-thyroidal extension; RLN, recurrent laryngeal nerve.

Data are expressed as n (%).

- Tumor location 1 was classified into upper, mid, and lower categories [13]. - Tumor location 2 was assessed for the invasion [14]. - Tumor location 3 was divided into three categories according to the angles between the tumor and the trachea for the trachea invasion risk stratification [15].

**Table 3:** Differences in ultrasonographic characteristics of the primary thyroid tumor.

	PTMC with lateral LN metastasis [N1b] (*n* = 90)	PTMC without lateral LN metastasis [N0] (*n* = 268)	*p*-value
Echogenicity			0.913
Marked hypoechoic	82 (91.1)	246 (91.8)	
Mild hypoechoic	7 (7.8)	18 (6.7)	
Isoechoic	1 (1.1)	4 (1.5)	
Hyperechoic	0 (0.0)	0 (0.0)	
Composition			0.180
Cystic	0 (0.0)	0 (0.0)	
Predominantly cystic	2 (2.2)	1 (0.4)	
Predominantly solid	1 (1.1)	7 (2.6)	
Solid	87 (96.7)	260 (97.0)	
Margin			0.087
Smooth	14 (15.6)	60 (22.4)	
Irregular	71 (78.9)	203 (75.7)	
Ill-defined	5 (5.5)	5 (1.9)	
Orientation			0.013
Parallel	18 (20.0)	91 (34.0)	
Non-parallel	72 (80.0)	177 (66.0)	
Calcification			0.053
No	6 (6.7)	19 (7.1)	
Punctate echogenic foci (microcalcification)	61 (67.8)	212 (79.1)	
Macrocalcification	22 (24.4)	33 (12.3)	
Rim calcification	1 (1.1)	4 (1.5)	
TIRAD			0.608
3 (low suspicion)	0 (0.0)	2 (0.8)	
4 (intermediate suspicion)	3 (3.3)	6 (2.2)	
5 (high suspicion)	87 (96.7)	260 (97.0)	

PTMC, papillary thyroid microcarcinoma; LN, lymph node; TIRAD, Thyroid Imaging Reporting and Data System.

Data are expressed as n (%).

Ultrasonographic findings of main thyroid tumor (echogenecity, composition, margin, orientation, and calcification) were evaluated based on the ACR TI-RADS [16-18].

#### 3.2.2 US for the thyroid gland and suspicious lymph node

US findings of background thyroid gland is summarized in **Supplementary Table 1**. There were no differences in echogenicity, sonographic feature, margin, and AP diameter between the two groups. US features for suspicious LN are summarized in **Supplementary Table 2**. Among patients with PTMC with lateral LN metastasis, the rate of suspicious LNs observed on both sides of the neck was 3.3%. The most frequently observed US feature of suspicious LN was echogenic foci (calcification), followed by cortical hyperechogenicity (55.6% and 24.4%, respectively). Cystic changes were observed in 12.2% of a total of 90 patients, and all of them showed no malignant cells on FNA and all of them showed no malignant cells on FNA. Washout fluid Tg was obtained and evaluated in all suspicious LN with cystic change and the range for washout fluid Tg was 9.70–659.0 ng/ml.

### 3.3 Predictors for lateral LN metastasis

Multivariate regression analysis was performed for both pathological and sonographic variables exhibiting significant differences. ETE, multifocality, upper lobe tumor location, and non-parallel shape were independent risk factors for lateral LN metastasis in patients with PTMC (**Table 4**).

**Table 4:** Risk factors for lateral LN metastasis in patients with papillary thyroid microcarcinoma.

	β	SE	OR (95% CI)	*p*-value
Tumor size	0.942	0.560	2.566 [0.856–7.697]	0.093
Extrathyroidal extension	1.599	0.436	4.948 [2.106–11.622]	**<0.001**
Multifocality	1.410	0.327	4.096 [2.160–7.768]	**<0.001**
Bilaterality	0.479	0.402	1.615 [0.735–3.550]	0.233
Location (upper)	1.165	0.309	3.207 [1.750–5.877]	**<0.001**
Orientation (nonparallel)	0.738	0.336	2.091[1.082–4.042]	**0.028**

LN, lymph node; SE, standard error; OR, odds ratio; CI, confidence interval.

The correlation with lateral LN metastasis in patients with papillary thyroid microcarcinoma, p<0.05.

### 3.4 Subgroup analysis to evaluate risk factors for skip metastasis in patients with lateral LN metastasis from PTMC

Among a total of 90 patients with lateral LN metastasis, 29 cases (32.2%) had skip metastasis (**Table 5**). There were no significant differences between skip metastasis and stepwise metastasis in age, sex, ETE, and the location of LN metastasis. However, a higher frequency of upper lobe tumor location was observed in skip metastasis compared to stepwise metastasis (55.2% vs. 31.1%, *p* = 0.029). Conversely, bilaterality and multifocality were more frequently found in the stepwise metastasis group (6.9% vs. 36.1%, *p* = 0.003, 24.1% vs. 54.1%, *p* = 0.008, respectively) and a greater number of metastatic LNs were observed in the stepwise metastasis group (2.9 ± 2.6 vs. 7.9 ± 4.7, *p* < 0.001).

**Table 5:** Clinicopathological findings between skip metastasis and stepwise metastasis in papillary thyroid microcarcinoma patients with lateral lymph node metastasis.

	Skip metastasis (*n* = 29)	Stepwise metastasis (*n* = 61)	*p*-value
Age (year)	47.5 ± 10.4	49.5 ± 12.5	0.447
Sex (female, %)	22 (75.9)	45 (73.8)	0.832
Tumor size (cm)	0.6 ± 0.2	0.7 ± 0.5	0.260
Tumor location (upper)	16 (55.2)	19 (31.1)	**0.029**
Bilaterality (%)	2 (6.9)	22 (36.1)	**0.003**
Multifocality (%)	7 (24.1)	33 (54.1)	**0.008**
Extrathyroidal extension (%)	7 (24.1)	16 (26.2)	0.832
Number of the involved LNs	2.9 ± 2.6	7.9 ± 4.7	**<0.001**
Location of LN metastasis^a^			
II (%)	13 (44.8)	28 (45.9)	0.706
III (%)	21 (72.4)	48 (78.7)	0.194
IV (%)	17 (58.6)	35 (57.3)	0.803

LN, lymph node.

^a^multiple LN metastasis was observed in the same patient.

Compared to stepwise metastasis, p<0.05.

The location of lateral LN metastasis was classified as belonging to level II (the upper jugular group), III (the middle jugular group), and IV( the lower jugular and medial supraclavicular group).

## 4 Discussion

This study focused on identifying risk factors for lateral LN metastasis in patients with PTMC. We evaluated clinicopathologic features and US findings not only in the main thyroid tumor but also in the background thyroid gland. Additionally, this study aimed to evaluate predictors of skip metastasis in patients with PTMC. The results revealed that ETE, tumor multifocality, upper lobe tumor location, and non-parallel tumor shape were independent predictors for lateral LN metastasis in patients with PTMC. Importantly, all these predictors can be assessed non-invasively using US, making it a valuable tool for risk stratification before the decision of AS.

PTC, the most common type of differentiated thyroid cancer, has seen a significant increase in incidence over the last few decades (22), resulting in considerable economic and emotional burden. As a result, AS is increasingly recommended as a suitable management option for low-risk PTC, particularly PTMC, given its favorable prognosis (23). The most important part of AS is to find appropriate criteria for AS, which could reduce the possibility of disease progression during observation. The definition of disease progression under AS is divided into two sections: tumor size growth and the emergence of new LN metastasis (14). The lower frequency of LN metastasis and the lack of significant benefit from prophylactic central LN dissection have supported lobectomy and even AS strategies in patients with PTMC. However, the presence of lateral LN metastasis can alter the treatment approach, with reported rates of up to 39.5% when prophylactic LN dissection is performed (24). This distinction can significantly impact the management strategy choice between AS and immediate surgery (IS) for patients with low-risk PTC. Moreover, LN metastasis has been linked to distant metastasis and recurrence in PTC patients, further emphasizing the importance of detecting and addressing LN involvement in the disease management (3, 25, 26). Our study demonstrated that patients with PTMC without LN metastasis (N0) at diagnosis showed no structural recurrence during an average follow-up period of 94.6 months, which could be safe candidates for AS.

In a Korean study analyzing 5,656 patients with PTMC, male gender was identified as one of the independent predictors for lateral LN metastasis, with the difference of loco-regional recurrence according to nodal stage (27). Similarly, a Japanese AS cohort study revealed that young age (<40 years) was an independent risk factor for newly developed LN metastasis among low-risk patients with PTMC (28). Young age and male gender are well-known predictors in patients with PTC; however, a Korean study demonstrated higher recurrence rates and mortality in male patients compared to female patients with PTC, but male gender was not an independent prognostic factor for recurrence in propensity score-matched patients with PTMC (29). In this study, we performed the PSM for age and sex among patients with PTMC; thus, the age and sex variables for the risk of LN metastasis could not be analyzed. Nevertheless, special attention is warranted to identify lateral LN metastasis in patients with young age and male gender.

In a Korean study analyzing 3,578 patients with PTMC, central LN metastasis, ETE, and multifocality were identified as significant risk factors for lateral LN metastasis (30). Our study also found similar trends, with multifocality and ETE being related to lateral LN metastasis, although central LN metastasis could not be evaluated due to the study’s design comparing N1b with N0. Many studies have shown that multifocality is a risk factor for LN metastasis in PTC and patients with PTMC (7, 31, 32), and there are still controversies regarding its relationship with prognosis. A recent study suggested that minimal ETE is linked to lateral LN metastasis (33), but there is no association between minimal ETE and recurrence among patients with PTMC (34). A future prospective study could explain the relationship between multifocality and clinical outcome in patients with PTMC.

At the time of the decision for AS in low-risk patients with PTMC, many histopathological risk factors are not readily available. However, tumor size, ETE, and multifocality can be evaluated by US with some limitations. For instance, capsular invasion on US can be defined as the percentage of the tumor perimeter in contact with the thyroid capsule, and PTC patients with >50% capsular invasion on US show a higher frequency of lateral LN metastasis (35). Loss of the echogenic capsule has been identified as a reliable US value for evaluating capsular invasion in PTC patients, with 75% sensitivity and 65% specificity, but a false discovery rate of 57.1% (36). US may have some limitations in accurately assessing multifocality due to the possibility of occult tumors with small size, and three patients were excluded because tumor was not observed in preoperative US of our study. Despite these challenges, our study showed the association of ETE and multifocality with lateral LN metastasis; thus, careful US evaluation of ETE and tumor number can provide valuable clues for the risk of lateral LN metastasis.

US findings play a critical role in predicting disease progression, especially lateral LN metastasis, in patients with PTMC considering AS because US should be performed in all patients before the decision of AS. Despite its importance, only a few studies have focused on US features for PTMC. A Chinese study highlighted that US features, such as central LN metastasis in the presence of concurrent HT, upper lobe tumor location, lack of a well-defined margin, and the presence of calcifications, were significantly associated with lateral LN metastasis in patients with PTMC (37). Another study identified upper lobe tumor location, microcalcification, and subcapsular lesions (defined as nodules abutting the thyroid capsule without intervening thyroid tissue) as factors associated with lateral LN metastasis (38). In the present study, we found that upper lobe tumor location and a non-parallel shape of the main thyroid tumor were independent predictors for lateral LN metastasis in patients with PTMC. The association between upper lobe tumor location and lateral LN metastasis has been consistently demonstrated in most studies focusing on patients with PTMC (39). Consequently, meticulous US follow-up is essential with a specific focus on detecting lateral LN metastasis in patients with PTMC with tumors located in the upper lobe. The upper lobe tumor location and PTMC were risk factors of skip metastasis (40, 41). This study evaluated risk factors of skip metastasis in patients with PTMC, and upper lobe tumor location was identified as a risk factor. Furthermore, skip metastasis could suddenly occur without other progressive signs including bilaterality, multifocality, and the expansion of metastatic LNs.

Apart from the main characteristics of the thyroid tumor, the background of the thyroid itself could also influence LN metastasis. The association between DTD and PTC has primarily been evaluated in the context of HT. Previous reports have indicated a higher prevalence of PTC in patients with HT compared to those without HT, and some studies have suggested a potential protective effect of HT in terms of recurrence and disease-related mortality of PTC (42). However, controversies still exist regarding this association. In our study, we found that coexisting HT, as determined by pathological results and US findings, showed no association with lateral LN metastasis in patients with PTMC. Similarly, other studies have shown no significant association between HT and LN metastasis or recurrence rate in patients with PTMC when HT was defined based on thyroid autoantibodies or pathology (43). Furthermore, a meta-analysis reported that there was no relationship between HT and LN metastasis in patients with PTMC and that HT had a negative association with LN metastasis in PTC cases larger than 1 cm (44).

This study has several limitations that need to be acknowledged. Firstly, the retrospective design of the study may introduce selection bias and limit the generalizability of the findings. Secondly, the evaluation of vascularity using Doppler US was not conducted due to the lack of data, which could have provided valuable information on the vascularity of the thyroid tumor and gland. Thirdly, age- and sex-matched data were used as controls (N0 group), which means that the effects of age and sex as potential risk factors were not specifically evaluated in this study. Despite these limitations, this study is valuable as it assessed and analyzed US findings of both the main tumor and the thyroid gland, allowing for the identification of potential candidates for AS without relying solely on pathological findings.

In conclusion, factors including upper lobe tumor location and non-parallel shape, along with ETE and multifocality, were identified as independent risk factors for lateral LN metastasis in patients with PTMC, and skip metastasis is also more commonly observed in tumors of the upper lobe. Therefore, meticulous US examinations to predict LN metastasis that include an assessment of tumor location, number, orientation shape, and the presence of ETE beyond mere detection of LN metastasis are necessary in the decision-making process for AS in especially when primary tumor was found in upper lobe.

## Data availability statement

The raw data supporting the conclusions of this article will be made available by the authors, without undue reservation.

## Ethics statement

The Institutional Review Board of Chonnam National University Hwasun Hospital (No. CNUHH-2020-271) approved this study. The studies were conducted in accordance with the local legislation and institutional requirements. Written informed consent for participation was not required from the participants or the participants’ legal guardians/next of kin in accordance with the national legislation and institutional requirements.

## Author contributions

JY: Data curation, Formal analysis, Investigation, Writing – original draft. JP: Data curation, Methodology, Writing – original draft. AH: Investigation, Software, Writing – review & editing. HK: Supervision, Validation, Writing – review & editing. H-CK: Conceptualization, Supervision, Writing – review & editing.
