# Corrigendum: Predictors of lateral lymph node metastasis and skip metastasis in patients with papillary thyroid microcarcinoma

**DOI:** 10.3389/fendo.2024.1480460

**Published:** 2024-09-02

**Authors:** Jee Hee Yoon, Ji Yong Park, A Ram Hong, Hee Kyung Kim, Ho-Cheol Kang

**Affiliations:** ^1^ Department of Internal Medicine, Chonnam University Medical School, Gwangju, Republic of Korea; ^2^ Division of Endocrinology and Metabolism, Department of Internal Medicine, Chonnam University Hwasun Hospital, Gwangju, Republic of Korea

**Keywords:** papillary thyroid microcarcinoma (PTMC), lymph node (LN), extra-thyroidal extension (ETE), upper lobe, non-parallel shape, multifocality

In the published article, there was a mistake in the Funding statement. The Funding statement for a grant from Chonnam National University Hwasun Hospital Institute for Biomedical Science was displayed as “HCRI 2019–01182”. The correct Funding statement appears below.

“The author(s) declare financial support was received for the research, authorship, and/or publication of this article. This research was supported by a grant (HCRI 20014) from the Chonnam National University Hwasun Hospital Institute for Biomedical Science.”

The authors apologize for this error and state that this does not change the scientific conclusions of the article in any way. The original article has been updated.

