# Supplementary material for: Predictors of lateral lymph node metastasis and skip metastasis in patients with papillary thyroid microcarcinoma
Source: Front Endocrinol (Lausanne). 2024 Jul 2;15:1392247. doi: 10.3389/fendo.2024.1392247 (PMC11250241; doi:10.3389/fendo.2024.1392247)
Supplement: Supplementary file 1 [file Table_1.docx]

**Supplementary Table 1. Ultrasonographic findings of thyroid parenchyma to evaluate diffuse thyroid disease.**

|  | PTMC with lateral LN metastasis [N1b]  (n= 90) | PTMC without lateral LN metastasis [N0]  (n=268) | *p*-value |
| --- | --- | --- | --- |
| Echogenicity |  |  | 0.389 |
| Isoechoic | 69 (76.7) | 193 (72.0) |  |
| Hypoechoic | 21 (23.3) | 75 (28.0) |  |
| Hyperechoic | 0 (0.0) | 0 (0.0) |  |
| Sonographic feature |  |  | 0.271 |
| Fine | 57 (63.3) | 152 (56.7) |  |
| Coarse/ Microlobulated | 33 (36.7) | 116 (43.3) |  |
| Margin |  |  | 0.211 |
| Smooth | 82 (91.1) | 254 (94.8) |  |
| Microlobulated | 8 (8.9) | 12 (4.5) |  |
| Macrolobulated | 0 (0.0) | 2 (0.7) |  |
| AP diameter |  |  | 0.300 |
| 1-2 cm (normal range) | 90 (100.0) | 261 (97.4) |  |
| <1 cm | 0 (0.0) | 3 (1.1) |  |
| >2 cm | 0 (0.0) | 4 (1.5) |  |

PTMC, papillary thyroid microcarcinoma; LN, lymph node; AP, antero-posterior.

Data are expressed as$n \left( \% \right).$

**Supplementary Table 2. Ultrasonographic findings of lymph nodes**

|  | PTMC with lateral LN metastasis [N1b]  (n= 90) |
| --- | --- |
| Location 1 |  |
| Left | 37 (41.1) |
| Right | 50 (55.6) |
| Both | 3 (3.3) |
| Location 2 |  |
| II | 5 (5.5) |
| III | 23 (25.6) |
| IV | 37 (41.1) |
| II, III | 5 (5.5) |
| III, IV | 10 (11.1) |
| II, III, IV | 10 (11.1) |
| Cystic change | 11 (12.2) |
| Echogenic foci (calcification) | 50 (55.6) |
| Cortical hyperechogenicity (focal/diffuse) | 22 (24.4) |
| Abnormal vascularity (peripheral/diffuse) | Not applicable |
| FNA |  |
| Not performed | 22 (24.4) |
| No malignant cells | 11 (12.2) |
| Suspicious LN metastasis | 57 (63.3) |

PTMC, papillary thyroid microcarcinoma; LN, lymph node; FNA, fine needle aspiration.

Data are expressed as$n\left( \% \right).$
